# Speyer Prize Competition

**Published:** 1906-04-07

**Authors:** 


### SPEYER PRIZE COMPETITION.

### STEWARD *v.* HOUSEKEEPER.

A Hospital Steward writes: —

I was pleased to see the outspoken criticism of "A Hospital Official" in the issue of The Hospital for the week ending March 31. I agree with nearly all the remarks made, especially do I endorse the last paragraph in which Mr. Hamilton's advocacy of a Housekeeper in preference to a Steward, in a Hospital containing beds of any number up to 300 is questioned. The best way to enlighten Mr. Hamilton on this point would be for him to visit some hospitals of the size he mentions. He would find, beyond a shadow of a doubt, that where there is not a Steward in name there is always a male officer under the Secretary who to all intents and purposes carries out the work of a Steward.

As far as my experience goes, it is very difficult to find a Housekeeper who is sufficiently versed in accounts and general business to enable her to carry out all the duties and responsibilities which must devolve upon the officer who receives and distributes the stores or to keep the elaborate accounts which Mr. Hamilton specifies as necessary.

I would point out also how very improbable it is that a thoroughly capable woman, who has gone through several years' training as a probationer, Nurse, and Sister, would give up her technical knowledge and skill in order to discharge the duties of a Housekeeper, for which her training as a nurse has not qualified her, and for which, under ordinary circumstances, the remuneration is so much less than she could obtain as a fully trained nurse. But, really, I should as soon expect efficiency in a London Police Court where there was a Magistrate and no Clerk as I should expect efficiency in a Hospital of 300 beds where there was a Secretary and no Steward.

Is it not common knowledge to Hospital Clerks that the many details they necessarily require for the strict keeping of their books are most difficult to get from women Housekeepers? Such an official may be a very good Housekeeper in the ordinary sense of the word, but it is seldom one finds a Housekeeper with any real knowledge of book-keeping, or capacity for it. There may be all the willingness in the world on the part of the Housekeeper to organise, to superintend, to preserve order in the servants' quarters (male and female), to prevent waste of water, gas, and electricity, but she has not the necessary qualifications. Again, it will be found that Stewards remain in the service of an Institution for a much longer time than do Housekeepers. If inquiries are made and the average is taken I dare venture to say that the terms of office held by Stewards as against that of Housekeepers will be at least double. We all know that in these days ladies are ambitious, and we cannot blame them if they chop and change about in their constant endeavour to advance their social status, and add to their income: at the same time this is bad for the Institutions.

In conclusion, I believe I am right in saying that until five or six years ago Westminster Hospital managed its affairs without a Resident Steward. The Secretary of that Institution paid some visits to Hospitals not unlike his own in size and he was struck by the improved internal methods of those Hospitals where a male Resident Steward was on duty. Surely this instance is a valuable comment on modern Hospital management as well as a blow at Mr. Hamilton's plea.

